# The *Legionella pneumophila* GIG operon responds to gold and copper in planktonic and biofilm cultures

**DOI:** 10.1371/journal.pone.0174245

**Published:** 2017-05-02

**Authors:** Kathleen Jwanoswki, Christina Wells, Terri Bruce, Jennifer Rutt, Tabitha Banks, Tamara L. McNealy

**Affiliations:** 1 Department of Biological Sciences, Clemson University, Clemson, South Carolina, United States of America; 2 Clemson Light Imaging Facility, Clemson University, Clemson, South Carolina, United States of America; Purdue University, UNITED STATES

## Abstract

*Legionella pneumophila* contaminates man-made water systems and creates numerous exposure risks for Legionnaires’ Disease. Because copper/silver ionization is commonly used to control *L*. *pneumophila*, its mechanisms of metal response and detoxification are of significant interest. Here we describe an *L*. *pneumophila* operon with significant similarity to the GIG operon of *Cupriavidus metallidurans*. The *Legionella* GIG operon is present in a subset of strains and has been acquired as part of the ICE-βox 65-kB integrative conjugative element. We assessed GIG promoter activity following exposure of *L*. *pneumophila* to multiple concentrations of HAuCl_4_, CuSO_4 and_ AgNO_3_. At 37°C, control stationary phase cultures exhibited GIG promoter activity. This activity increased significantly in response to 20 and 50uM HAuCl_4_ and CuSO_4_ but not in response to AgNO_3_. Conversely, at 26°C, cultures exhibited decreased promoter response to copper. GIG promoter activity was also induced by HAuCl_4_ or CuSO_4_ during early biofilm establishment at both temperatures. When an *L*. *pneumophila* GIG promoter construct was transformed into *E*. *coli* DH5α, cultures showed baseline expression levels that did not increase following metal addition. Analysis of *L*. *pneumophila* transcriptional regulatory mutants suggested that GIG up-regulation in the presence of metal ions may be influenced by the stationary phase sigma factor, RpoS.

## Introduction

*Legionella pneumophila*, the etiological agent of Legionnaires’ Disease (LD), is the leading cause of bacterial waterborne disease outbreaks in the United States [1]. This Gram-negative bacterium is ubiquitous in both natural and man-made aquatic environments, where it replicates as an intracellular parasite of free-living protozoa [2]. Most cases of LD can be traced back to human-made aquatic systems with above-ambient water temperatures: cooling towers, hot water heaters, fountains, and air conditioning units have all served as sources of outbreaks [2].

*Legionella* persist as part of the biofilm community in human-made aquatic systems, and these systems are routinely treated to inhibit microbial growth. Chlorine and chloramine are the most common disinfectants used in the US. Other treatments include chlorine dioxide, UV radiation, hyperchlorination, bromine, and copper/silver ionization. While such treatments work well against fecal coliforms and other bacteria that enter the system from outside sources, they are less effective at eliminating pathogens like *Legionella* that exist within resistant biofilms of the system itself. Copper/silver ionization is often used to control *Legionella* in large recirculating water systems, particularly industrial plumbing, but its effectiveness is controversial [3–6]. Some studies indicate that high levels of copper inhibit *Legionella* growth and survival, but others have demonstrated increased persistence of *Legionella* in biofilms formed on copper [7]. *Legionella* also demonstrates increased resistance to copper at lower temperatures [8–9].

Small amounts of copper are required for mitochondrial electron transport and other enzymatic reactions, but high intracellular copper levels are considered toxic to most prokaryotes [10–12]. General mechanisms of bacterial metal resistance include export across the plasma membrane, sequestration by binding proteins, and reduction to a less toxic state. While *Legionella* is sensitive to elevated concentrations of numerous metals [13], few of its metal resistance mechanisms have been described. A *Legionella* copper-translocating PIB-type ATPase (CopA) was shown to confer copper resistance when expressed in a copper-sensitive *E*. *coli* strain [14], and the *helABC* locus was reported to encode three proteins involved in heavy metal resistance and cytopathogenicity [15]. Additional mechanisms by which *Legionella* sense and respond to metal ions in their environment remain to be characterized.

Here we describe a *Legionella pneumophila* operon (*lpg2105-2108)* with significant homology to the “gold induced genes” (GIG) operon of *Cupriavidus metallidurans* [16]. The operon appears to have been acquired by a subset of *L*. *pneumophila* strains as part of the horizontally-transferred ICE-βox integrative conjugative element [17]. In planktonic cultures, promoter expression occurs at 37°C in response to gold and copper, but at 26°C it occurs only in response to growth phase. Under biofilm conditions, promoter response to metal ions is seen at both temperatures.

## Material and methods

### Identification of the *L*. *pneumophila* GIG operon and homologous operons

The *L*. *pneumophila* GIG operon was originally identified through a BlastP search of proteins from the *L*. *pneumophila* subsp. *pneumophila* str. Philadelphia 1 genome (NCBI NC_002942.5), using the four *C*. *metallidurans* GIG proteins as queries (Table 1). Additional homologous operons in *L*. *pneumophila* were subsequently identified through BlastP searches of five sequenced *L*. *pneumophila* subsp. *pneumophila* strains, using the *L*. *pneumophila* GIG proteins as queries. Hits with a query cover of at least 80% and an E-value of less than 1.0E^-15^ were retained in this analysis. Homologous operons were also identified in the genomes of the *Francisella tularensis* (NC_006570.2), *Burkholderia pseudomallei* (NC_012695.1), *Polaramonas* sp. JS666 (NC_007948.1) and *Pseudomonas fluorescens* (NC_007492.2) using similar methods. Predicted protein sequences from each operon were concatenated and aligned with MAFFT v.7 [18]. A maximum likelihood tree was constructed in PhyML using the LG amino acid substitution model and the SPR method of topology estimation [19]. Reliability of the tree was assessed with 500 bootstrap replicates, and branches reproduced in fewer than 50% of the replicates were collapsed.

**Table 1:** Similarity and identity of gold induced genes (GIG) operon in *L*. *pneumophila* Philadelphia 1 to *C*. *metallidurans* GIG operon.

*C*. *metallidurans* GIG protein	Length (aa)	*L*. *pneumophila* Philadelphia 1 GIG protein	Length (aa)	E-value	Query cover (%)	Identity (%)	Annotation information
Rmet_4682 (ABF11544.1)	156	lpg2105 (YP_096118.1)	165	1.00E-19	85	38	Predicted inner membrane protein with a DoxX domain (PF07681)
Rmet_4683 (ABF11545.1)	258	lpg2106 (YP_096119.1)	259	8.00E-16	89	22	Contains N-terminal DUF2063 domain with putative role in DNA binding and transcriptional regulation
Rmet_4684 (ABF11546.1)	278	lpg2107 (YP_096120.1)	284	7.00E-72	98	40	DUF692 family of uncharacterized bacterial proteins; possibly involved in methanobactin synthesis
Rmet_4685 (ABF11547.1)	94	lpg2108 (YP_096121.1)	97	9.00E-06	81	33	DUF2282 family of putative integral membrane and signal proteins

### Strains and media

In all experiments, wild type *L*. *pneumophila* strain Lp02 was cultured at 37°C or 26°C for three days on buffered charcoal yeast extract agar with 100μg/ml thymidine (BCYE-T). *L*. *pneumophila* Lp02 is a derivative of *L*. *pneumophila* Philadelphia 1 and contains the complete ICE-βox region. Lp02 *ΔletA* and *ΔrpoS* mutants were grown on BCYE with 20μg/ml kanamycin. All *Legionella pneumophila* strains were provided by Michelle Swanson (University of Michigan). *Escherichia coli* DH5α was grown at 37°C or 26°C for 24 hours on Tryptic Soy Agar (TSA). Broth cultures consisted of ACES (*N*-(2-Acetamido)-2-aminoethanesulfonic acid)-buffered yeast extract (AYE) for *Legionella* strains (with antibiotics and thymidine as necessary) and Tryptic Soy Broth (TSB) for *E*. *coli* strains.

#### Reverse Transcriptase-PCR

Total RNA was isolated from stationary phase cultures using the Promega SV RNA Isolation kit. RNA was DNase treated to ensure removal of contaminating DNA prior to RT-PCR analysis. Three sets of primers spanning the four genes of the operon (2105–2106; 2106–2107; 2107–2108) were used to confirm the single transcript containing the four genes. The Verso 1-step Reverse Transcriptase PCR kit was used following manufacturer’s instructions. Reactions were carried out in 25ul final volume using each primer set and 15ng of RNA per reaction. Amplified fragments were analysed on a 1.0% agarose gel.

### Construction of pGIG reporter gene vector

The *flaA* promoter of *pflaA* (a GFP reporter gene vector provided by M. Swanson, [20]), was replaced with 180nt of the upstream *lpg2105-2108* predicted promoter region using standard cloning methods. The 180nt upstream of *lpg2105-2108* represents the intergenic region between *lpg2108* and *lpg2109*. Bacterial promoter regions have been shown to be enriched in the intergenic regions of the genome and depleted from coding regions [21]. The entire intergenic region was cloned in order to capture as many potential promoter binding sites as possible for subsequent experiments. The resulting plasmid, *pGIGgfp*, was transformed into *L*. *pneumophila* Lp02, *E*. *coli* DH5α, *L*. *pneumophila* Lp02 *ΔletA*, and *L*. *pneumophila* Lp02 *ΔrpoS*.

### Reporter gene activity in planktonic cultures

The effect of metal ions on *L*. *pneumophila* growth kinetics and *pGIGgfp* reporter gene activity were measured by incubating planktonic cultures for 72 hours at 150rpm in the presence or absence of gold, copper or silver ions. Cultures were incubated at 37°C with 20μM or 50μM of gold chloride (HAuCl_4_), 20μM or 50μM copper sulfate (CuSO_4_), or 50μM or 150μM silver nitrate (AgNO_3_), or no additional ions (control). Cultures were incubated at 26°C with 5μM and 10μM HAuCl_4_, 200μM and 275μM CuSO_4_, or no additional ions (control). Every three hours, absorbance (OD_600_) was measured using a Genesys 6 spectrophotometer, and GFP fluorescence (485 nm excitation/528 nm emission) was measured using a Biotek Synergy H1 plate reader.

The effect of metal ions on *E*. *coli* growth kinetics and *pGIGgfp* reporter gene activity was measured by incubating planktonic cultures for 24 hours at 150rpm in the presence or absence of gold or copper. Cultures were incubated at 37°C with 20μM or 50μM HAuCl_4_, 20μM or 50μM CuSO_4_, or no additional ions. Absorbance and GFP fluorescence were measured hourly as described above.

All experiments were performed in triplicate. Data were normalized by dividing the GFP relative light units (RLU) by the OD_600_. The effects of metal ion, ion concentration, growth phase, and their interactions on the magnitude of GFP fluorescence were each analyzed separately.

### Reporter gene activity in biofilms

*L*. *pneumophila* Lp02 *pGIGgfp* biofilms were established as previously described [22–23]. Briefly, bacteria were inoculated into glass petri dishes containing glass slides in 10% AYE solution and incubated for 24 hours, then transferred to 100% AYE for the remainder of the incubation. Cultures were incubated at 26°C or 37°C with 20μM HAuCl_4_, 20μM CuSO_4_, or no additional ions. Biofilms were grown for 120 hours and assessed for GIG activity at 24, 48, 72, 96, and 120h. At each time point, biofilms were washed twice with sterile ultrapure water (UPW) to remove non-attached bacteria. Slides were aseptically removed, briefly air dried, and fixed in paraformaldehyde for 10 minutes. Slides were rinsed with UPW and dried; then coverslips were mounted using a 50/50 v/v solution of glycerol:phosphate buffered saline (1X PBS). All experiments were performed in triplicate.

### Image analysis

Biofilms were imaged using a Leica SPE spectral confocal microscope (63X, oil immersion objective, NA = 1.30; Leica Microsystems, Buffalo Grove, IL) in the Clemson Light Imaging Facility. Three DIC (differential interference contrast) images and three corresponding GFP images were obtained for each slide. For assessment of promoter activity, ImageJ software and DIC images were used to generate regions of interest (ROIs) corresponding to individual bacteria within the biofilm. The ROIs were outlined and numbered, and their areas were measured. A binary image “mask” of the ROIs was used as an overlay on the corresponding GFP image. The signal intensity within each ROI on the GFP image was measured and used as an indication of GFP expression in each bacterium (ROI). All biofilm samples produced some level of green fluorescence. To account for this primary fluorescence, the intensity of control biofilms was determined and gated out (subtracted) from the intensity of treated biofilms to determine GFP expression due solely to the addition of gold or copper. The 37°C control fluorescence intensity value was applied to all samples. Treatment effects were assessed using Z-scores with a 99% confidence interval.

To measure the biofilm biomass that demonstrated GIG activity, confocal images from each time point were analyzed using COMSTAT software [24]. Bio-volume was estimated from calculations of biofilm biomass, which were based on the number of bacteria-containing pixels in all images of a stack, multiplied by voxel size and divided by the stack’s substratum area. A one way analysis of variance (ANOVA) was used to compare bio-volumes among biofilms and time points. A significance level of *p*<0.05 was used for all tests.

## Results

### Description of the *L*. *pneumophila* GIG operon

The *L*. *pneumophila* GIG operon spans 2421 bases from 2352473 to 2354894 in the *L*. *pneumophila* subsp. *pneumophila* str. Philadelphia 1 genome (NC_002942.5). It is located within the ICE-βox, a 65-kB integrative conjugative element found in approximately 18% of surveyed *Legionella* strains and associated with increased virulence and oxidative stress tolerance [17, 25]. The operon contains four open reading frames encoding proteins of unknown function (*lpg2105-lpg2108*).

The first *L*. *pneumophila* GIG gene, *lpg2105*, encodes a 165-aa predicted inner membrane protein with a DoxX domain (PF07681). DoxX proteins exhibit some similarity to the thiosulphate:quinone oxidoreductase small subunit, DoxD, but their precise function is unknown [26]. The second GIG gene, *lpg2106*, encodes a 259-aa protein with an N-terminal DUF2063 domain that is predicted to function in DNA binding and transcriptional regulation [27]. The third GIG gene, *lpg2107*, encodes a 284-aa protein assigned to the DUF692 family of uncharacterized bacterial proteins. Other members of this family are key enzymes in the biosynthesis of methanobactins, secreted copper-binding and copper-reducing peptides produced by a variety of bacteria [28]. The final gene, *lpg2108*, encodes a small 97-aa protein assigned to the DUF2282 family of putative integral membrane and signal peptide proteins.

### Characterization of homologous operons

The *L*. *pneumophila* str. Philadelphia 1 genome also contains three additional regions with significant sequence similarity to the GIG operon, designated here as homologous operons H2, H3 and H4 (Table 2). The H2 operon (*lpg0665-lpg0669*) is structurally similar to the GIG operon and contains homologs of all four GIG genes, arranged in an identical order and strand orientation. The H3 operon (*lpg2253-lpg2255*) lacks an *lpg2105* homolog and therefore contains only three of the four GIG genes, again arranged in identical order and strand orientation. The H4 operon (*lpg0671- lpg0676*) contains homologs of all four GIG genes, but in a different order and on different strands. The first H4 gene (*lpg0671*) shares a DoxX domain with *lpg2105* but encodes a much larger NADH dehydrogenase transmembrane protein. Two additional genes are also present: an acetoacetate decarboxylase gene (*lpg0672*) and an adenylate cyclase gene (*lpg0674*). Rather than an operon, H4 may be better described as a cluster of genes that includes the four GIG homologs. They are unlikely to be transcribed as a unit, given their differing strand orientations.

**Table 2:** Homologous operons in *L*. *pneumophila* genomes to *lpg2105-2108* GIG operon.

	GIG		H2		H3		H4	
Strain	Locus tag	Start position and strand	Locus tag	Start position and strand	Locus tag	Start position and strand	Locus tag	Start position and strand
**Philadelphia 1**	lpg2105	2352970 (-)	lpg0665	716610 (-)			lpg0671	719987 (+)
**NC_002942.5**	lpg2106	2353739 (-)	lpg0666	717386 (-)	lpg2253	2557140 (-)	lpg0675	725484 (-)
	lpg2107	2354586 (-)	lpg0667	718248 (-)	lpg2254	2558033 (-)	lpg0676	726280 (-)
	lpg2108	2354894 (-)	lpg0669	718508 (-)	lpg2255	2558333 (-)	lpg0673	723047 (+)
**Paris**			LPP_RS03625	801134 (-)			LPP_RS03650	804510 (+)
**NC_006368.2**			LPP_RS03630	801910 (-)	LPP_RS11155	2543886 (-)	LPP_RS03670	809982 (-)
			LPP_RS03635	802772 (-)	LPP_RS11160	2544746 (-)	LPP_RS03675	810802 (-)
			LPP_RS03640	803032 (-)	LPP_RS11165	2545073 (-)	LPP_RS03660	807569 (+)
**Corby**			LPC_RS03790	827443 (-)			LPC_RS03815	830819 (+)
**NC_009494.1**			LPC_RS03795	828003 (-)	LPC_RS11785	2673120 (-)	LPC_RS03835	836292 (-)
			LPC_RS03800	829080 (-)	LPC_RS11790	2673980 (-)	LPC_RS03840	837112 (-)
			LPC_RS03805	829340 (-)	LPC_RS11795	2674376 (-)	LPC_RS03825	833879 (+)
**Lens**			LPL_RS03540	790101 (-)			LPL_RS03565	793477 (+)
**NC_006369.1**			LPL_RS03545	790877 (-)	LPL_RS11000	2494966 (-)	LPL_RS03585	798950 (-)
			LPL_RS03550	791739 (-)	LPL_RS11005	2495826 (-)	LPL_RS03590	799770 (-)
			LPL_RS03555	791999 (-)	LPL_RS11010	2496153 **(-)**	LPL_RS03575	796537 (+)
**Alcoy**			LPA_RS03750	813482 (-)			LPA_RS03775	816859 (+)
**NC_014125.1**			LPA_RS03755	814258 (-)	LPA_RS11585	2646878 (-)	LPA_RS03795	822332 (-)
			LPA_RS03760	815120 (-)	LPA_RS11590	2647738 (-)	LPA_RS03800	823152 (-)
			LPA_RS03765	815380 (-)	LPA_RS11595	2648134 (-)	LPA_RS03785	819919 (+)

**Fig 1:**
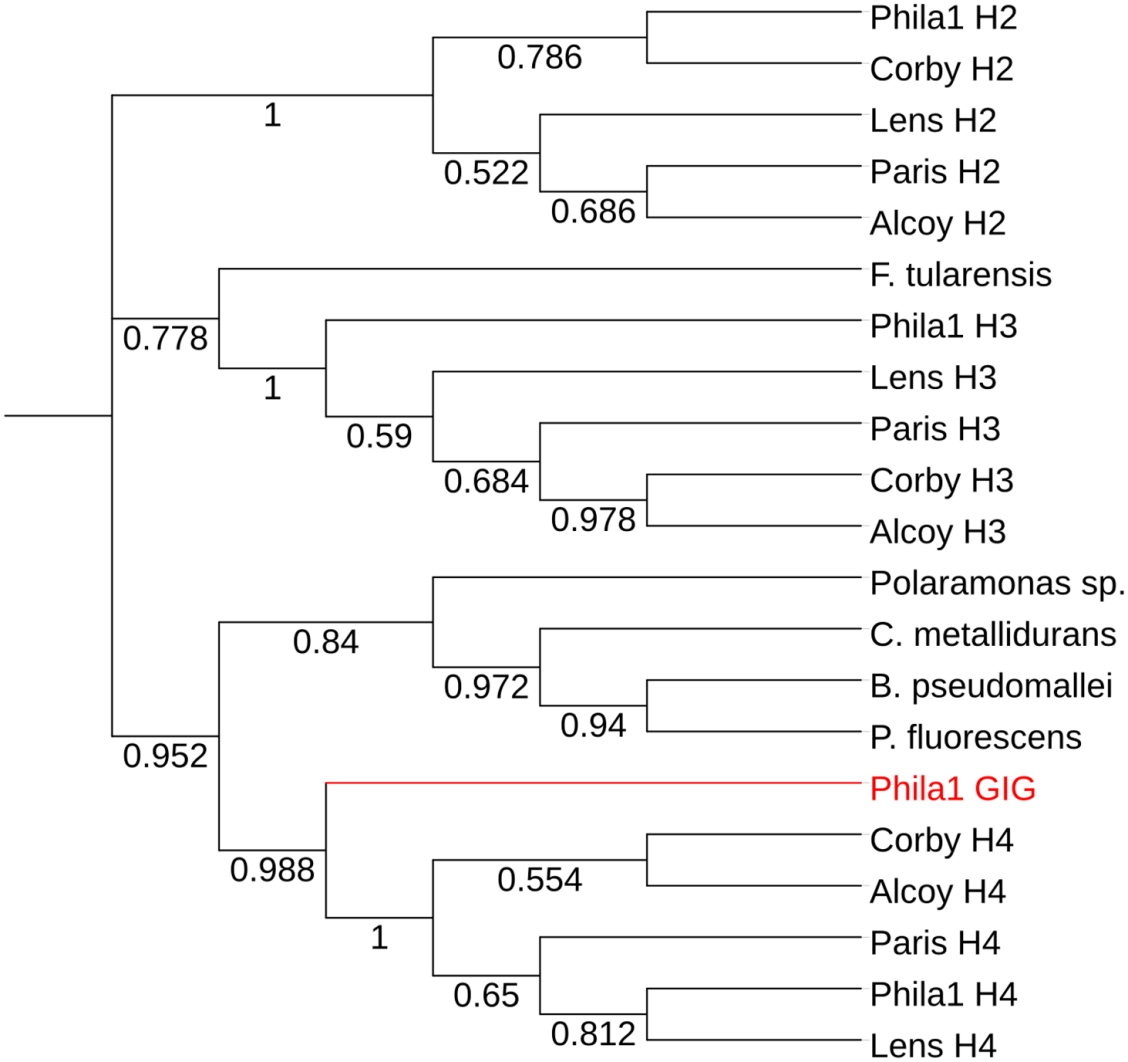
Maximum likelihood phylogeny for the GIG operon showing the relationship of *L*. *pneumophila lpg2105-2108* to 4 additional *Legionella* species and five other bacterial species. Numbers over branches show bootstrap support values (500 replicates).

We surveyed the genomes of four additional *L*. *pneumophila* strains for the presence of the GIG, H2, H3 and H4 operons (Table 2). While all strains contained copies of H2, H3 and H4, the GIG operon was present only in *L*. *pneumophila* Philadelphia 1. This is consistent with its acquisition as part of the ICE-βox. A maximum likelihood phylogenetic tree indicated that the *L*. *pneumophila* GIG operon was most similar to the *C*. *metallidurans* GIG operon, as well as to number of homologous operons from multiple environmental and/or pathogenic bacteria (Fig 1).

### *lpg2105-2108* operon

**Fig 2:**
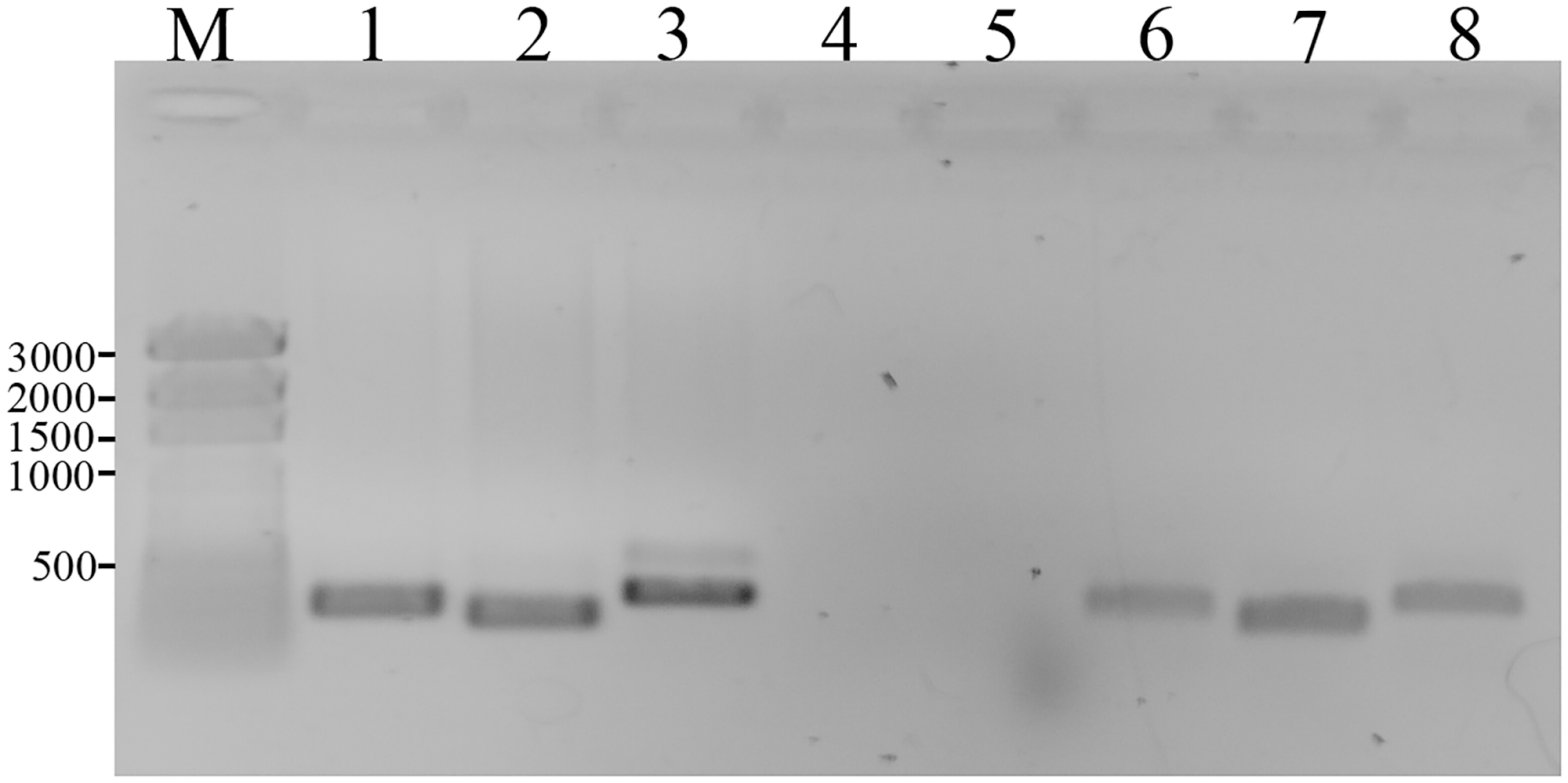
Reverse Transcriptase PCR using gene spanning primers. M-Marker; 1: positive control 2105F/2106R primers; 2: positive control 2106F/2107R primers; 3: positive control 2107F/2108R primers; 4: no template control; 5: no RT control; 6: RNA 2105F/2106R primers; 7: RNA 2106F/2107R primers; 8: RNA 2107F/2108R primers.

The four genes–*lpg2105-2108* –are predicted to be transcribed as a single unit based on genomic analysis. Using reverse transcriptase PCR, we confirmed that the genes are co-transcribed. Using three sets of primers spanning the end of one gene and the beginning of the next, RT-PCR reactions for gene combinations 2105–2106, 2106–2107 and 2107–2108 were positive (Fig 2).

### GIG promoter activity in planktonic cultures

Expression of the *C*. *metallidurans* GIG operon is influenced by the presence of gold and copper ions in the growth medium [16]. To test whether the *L*. *pneumophila* GIG operon was similarly affected, we created a GFP reporter construct whose expression was driven by the 180nt upstream promoter region of the *L*. *pneumophila* GIG operon. We measured GFP fluorescence to monitor expression of the *pGIGgfp* reporter in *L*. *pneumophila* Lp02 exposed to multiple concentrations of gold, copper, and silver ions. At both 37° and 26°C, we observed an extended lag phase in a concentration dependent manner when HAuCl_4_ or CuSO_4_ was added to the cultures. Cultures grown in the presence of 50μM of gold or copper never reached the same maximum OD_600_ as controls.

To account for the individual growth rates of each culture, mid-exponential (ME) and late stationary (LS) growth phases were identified in individual cultures, and the promoter activity was compared at each growth phase. Late stationary phase was defined as the time point prior to decline of OD_600_ that preceded death of the culture. GFP was normalized to OD_600_ at each time point to accurately compare promoter activity across cultures.

**Fig 3:**
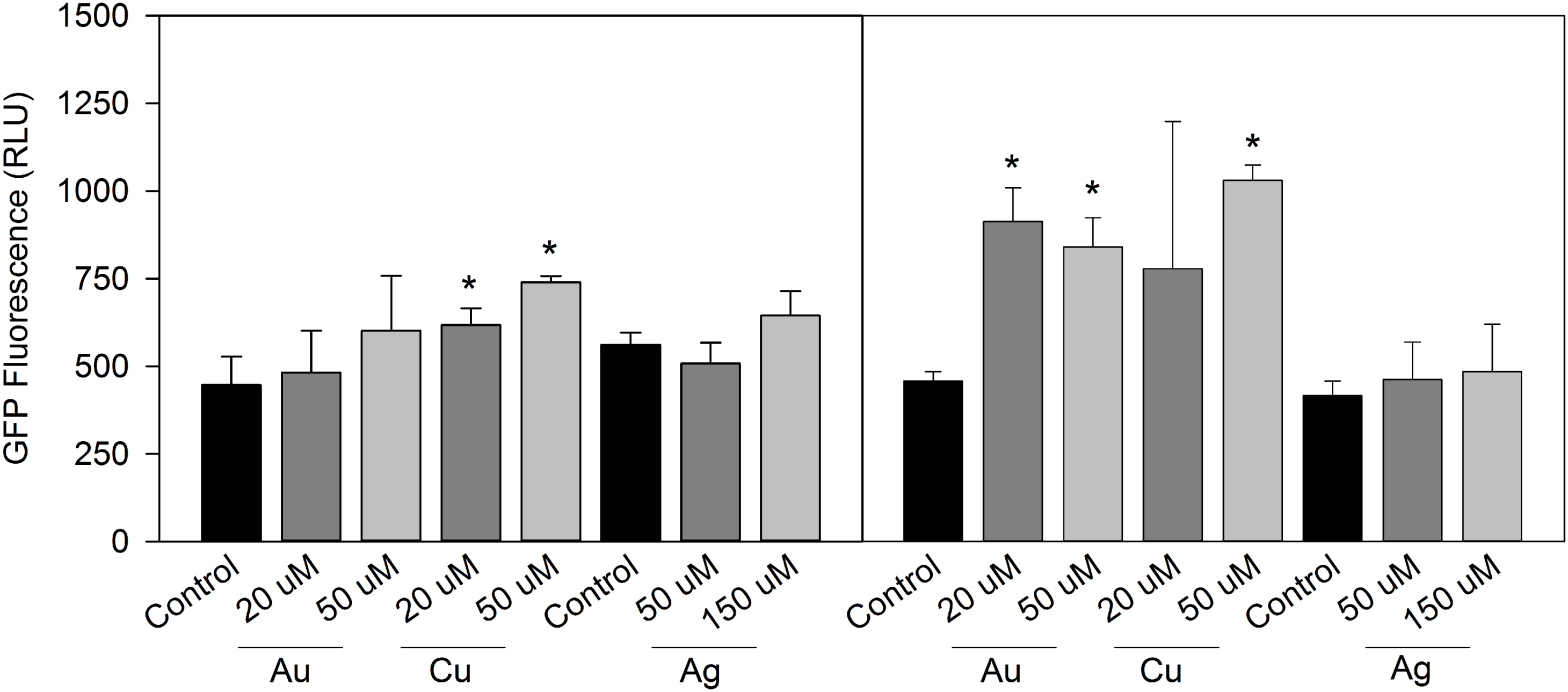
Effect of gold, copper and silver on *pGIGgfp* activity in *L*. *pneumophila* at 37°C. GIG operon activity measured by GFP expression of *pGIGgfp* in control media, with 20μM HAuCl_4_, 50μM HAuCl_4_, 20μM CuSO_4_ and 50μM CuSO_4_ at mid-exponential (ME) and late stationary (LS) growth phases (a). GFP expression in response to 50μM and 150μM AgNO_3_ (b). Data presented from three independent experiments ± SD (* = p < 0.05).

At 37°C, control cultures of *L*. *pneumophila* Lp02 *pGIGgfp* showed similar levels of *lpg2105-2108* promoter activity across all growth phases. The addition of 50μM AgNO_3_ did not affect culture growth or induce a promoter response. 150μM AgNO_3_ inhibited culture growth but did not induce a promoter response (Fig 3). The promoter response to HAuCl_4_ and CuSO_4_ differed between growth phases. At the mid-exponential growth phase, only CuSO_4_ treatment induced moderate promoter expression. At the late stationary growth phase, both HAuCl_4_ and CuSO_4_ induced higher levels of promoter expression (Fig 3).

At 26°C, *L*. *pneumophila* Lp02 growth was inhibited by the 20μM HAuCl_4_ used at 37°C, and HAuCl_4_ concentrations were therefore reduced to 5μM and 10μM. *Legionella* demonstrates increased resistance to copper at lower temperatures [9], and 26°C cultures showed no inhibition to the 20μM and 50μM CuSO_4_ used at 37°C. CuSO_4_ concentrations were therefore increased to 200μM and 275μM at 26°C to assess the promoter response under similar growth kinetics.

**Fig 4:**
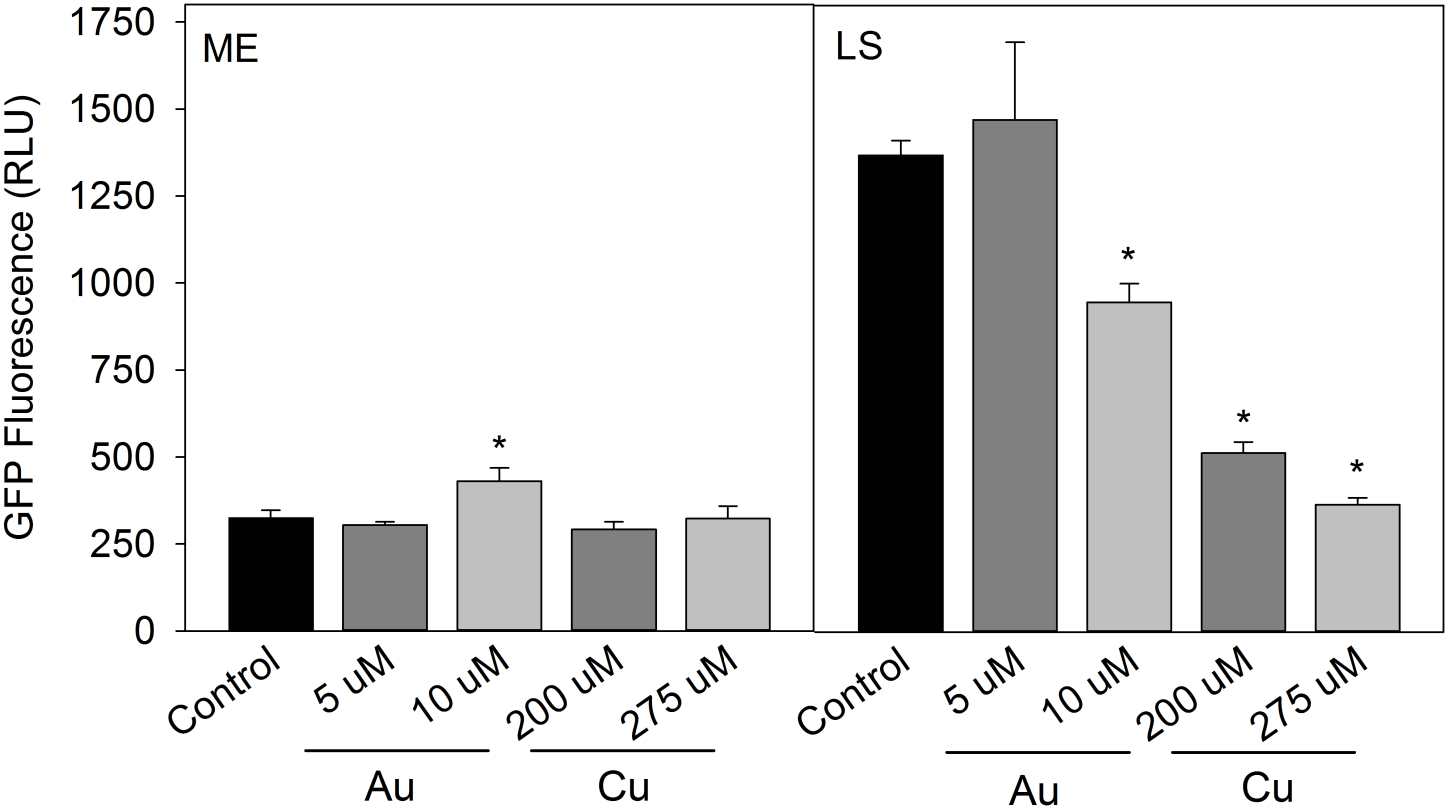
Effect of gold and copper on *pGIGgfp* activity in *L*. *pneumophila* at 26°C. GIG operon activity measured by GFP expression of *pGIGgfp* in control media, with 20μM HAuCl_4_, 50μM HAuCl_4_, 20μM CuSO_4_ and 50μM CuSO_4_ at mid-exponential (ME) and late stationary (LS) growth phases. Data presented from three independent experiments ± SD (* = p< 0.05).

The effect of growth phase on promoter expression was more pronounced at 26°C than at 37°C. Control cultures showed a significant increase in promoter activity at LS phase, a result which was not observed at 37°C and that suggested temperature regulation of the operon. 10μM HAuCl_4_ caused a modest increase in promoter activity at ME but reduced the level of promoter activity at LS. The addition of copper caused a significant decrease in promoter activity at LS (Fig 4).

**Fig 5:**
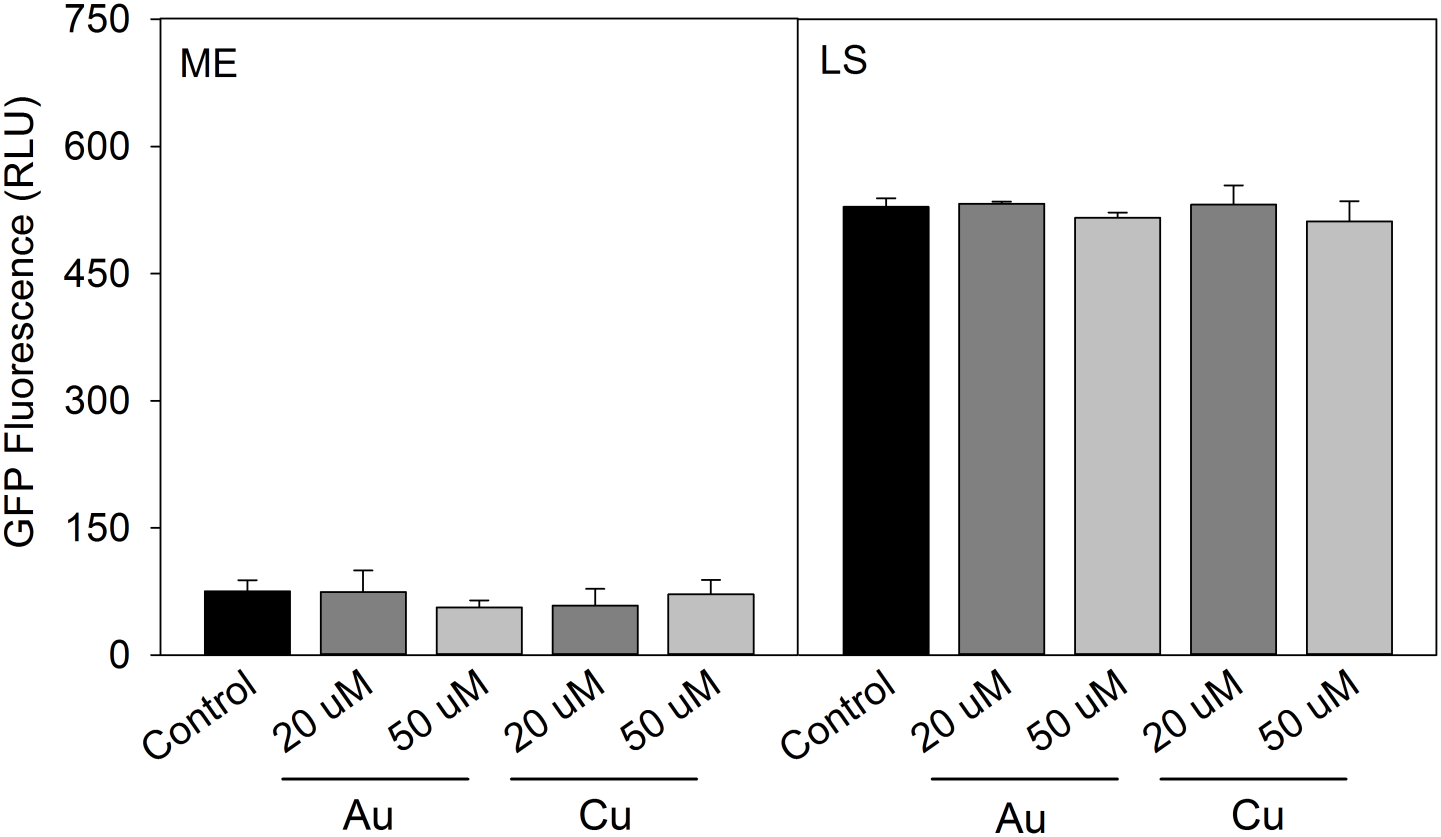
Effect of gold and copper on *pGIGgfp* activity in *E*. *coli* at 37°C. GIG operon activity measured by GFP expression of *pGIGgfp* in control media with addition of with 20μM HAuCl_4_, 50μM HAuCl_4_, 20μM CuSO_4_ or 50μM CuSO_4_ at mid-exponential (ME) and late stationary (LS) growth phases. Data presented are from three independent experiments ± SD.

The complex promoter response to temperature, growth phase and metal addition suggests that multiple levels of operon regulation interact in planktonic culture. To further investigate mechanisms of operon regulation, we examined promoter expression in *E*. *coli* DH5α transformed with *pGIGgfp* as well as in two *L*. *pneumophila* mutants in the major stationary phase regulation pathways. *E*. *coli* DH5 α, which does not possess the operon, showed no change in growth kinetics in the presence of HAuCl_4_ or CuSO_4_. At neither 37°C nor 26°C did *E*. *coli* show promoter activity to gold or copper (Fig 5). These results suggest a unique regulator in *L*. *pneumophila* for metal induction of the GIG operon. Significant increase in promoter expression in response to HAuCl_4_ and CuSO_4_ was seen during stationary phase, and we therefore investigated known regulators of *L*. *pneumophila* stationary phase gene expression. Mutants for the stationary phase regulators LetA and RpoS were transformed with *pGIGgfp* to assess their potential involvement.

The *Legionella* two-component regulatory system, LetA/S, induces the expression of virulence and transmission traits at stationary phase [29–30]. Lp02 *ΔletA pGIGgfp* cultures showed delayed entry into exponential phase when exposed to HAuCl_4_ or CuSO_4_, similar to that seen in wild type Lp02 *pGIGgfp* cultures. Lp02*ΔletA* cultures with added HAuCl_4_ or CuSO_4_ exhibited greater promoter activity compared to Lp02 *ΔletA* cultures with no metal addition. The magnitude of metal-induced promoter up-regulation was greater at the LS phase. However, wild type and *ΔletA* cultures responded similarly to metal addition: there were no significant differences in promoter activity between Lp02 *ΔletA* cultures and wild type Lp02 cultures at the same metal concentration.

**Fig 6:**
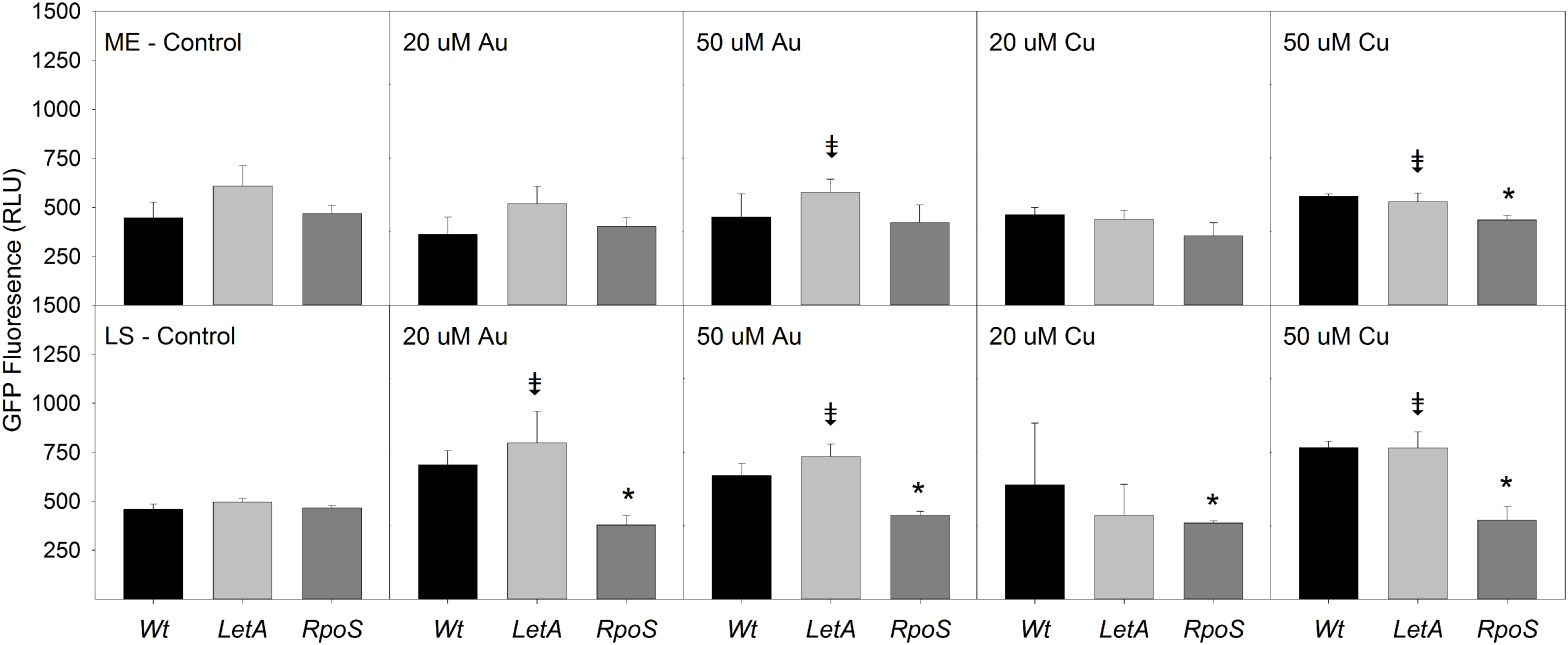
Effect of gold and copper on *pGIGgfp* activity *L*. *pneumophila* Lp02 *ΔletA* and *ΔrpoS*. GIG operon activity measured by GFP expression of *pGIGgfp* in control media with addition of with 20μM HAuCl_4_, 50μM HAuCl_4_, 20μM CuSO_4_ or 50μM CuSO_4_ at mid-exponential (ME) and late stationary (LS) growth phases. Data presented are from three independent experiments ± SD. (* = significantly different from WT with same metal addition in same panel; p < 0.05, † = significantly different from mutant with no metal addition in first panel; p<0.05)

Lp02 *ΔrpoS pGIGgfp* cultures exhibited an extended lag phase in response to HAuCl_4_ or CuSO_4_, and 50μM CuSO_4_ cultures showed increased promoter expression at ME phase. The promoter response to metal addition was dampened in Lp02 *ΔrpoS pGIGgfp* stationary phase cultures: mutant cultures showed reduced promoter activity compared to wild type in response to both HAuCl_4_ and CuSO_4_ addition, suggesting that RpoS may play a role in promoter expression, particularly at stationary phase (Fig 6).

### GIG promoter activity in biofilms

**Fig 7:**
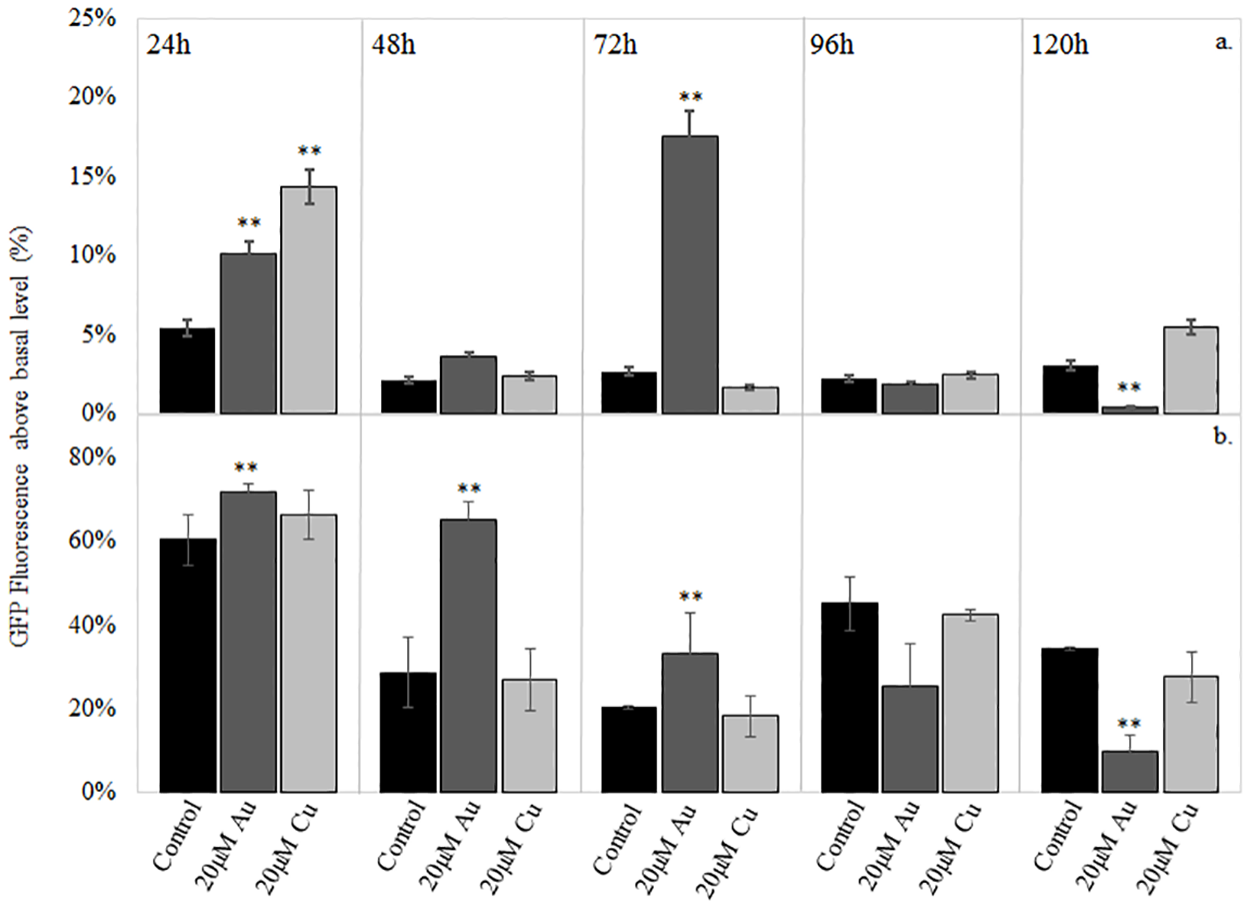
Analysis of pGIGgfp expression during biofilm establishment and development at a) 26°C in *L*. *pneumophila* control biofilms and with the addition of 20μM HAuCl4 or 20μM CuSO4 and at b) 37°C in *L*. *pneumophila* control biofilms and with the addition of 20μM HAuCl4 or 20μM CuSO4. Percent GFP intensity above basal level (determined at 26°C) is shown. Data presented are from three independent experiments ±SE (p<0.05)

*L*. *pneumophila* primarily persists in the environment as a biofilm [31–33]. To assess operon activity under more natural growth conditions, Lp02 *pGIGgfp* biofilms were established and grown at two temperatures in the presence and absence of 20μM HAuCl_4_ or CuSO_4_. Analysis of biofilms grown at 26°C revealed that promoter activity increased significantly in early phase biofilms when HAuCl_4_ or CuSO_4_ was added (Fig 7a). At 24h, a significant increase in GIG expression in response to both HAuCl_4_ and CuSO_4_ was observed. At 72h, an additional increase was observed in response to HAuCl_4_ exposure but not to CuSO_4_. The 26°C biofilms did not exhibit the increased sensitivity to gold as seen in the planktonic cultures.

Similar to planktonic cultures, greater promoter expression was measured in 37°C biofilms than in 26°C (Fig 7b). Increased promoter expression was seen in early phase biofilms in response to gold and copper at 24h and in response to gold at 48h and 72h.

**Fig 8:**
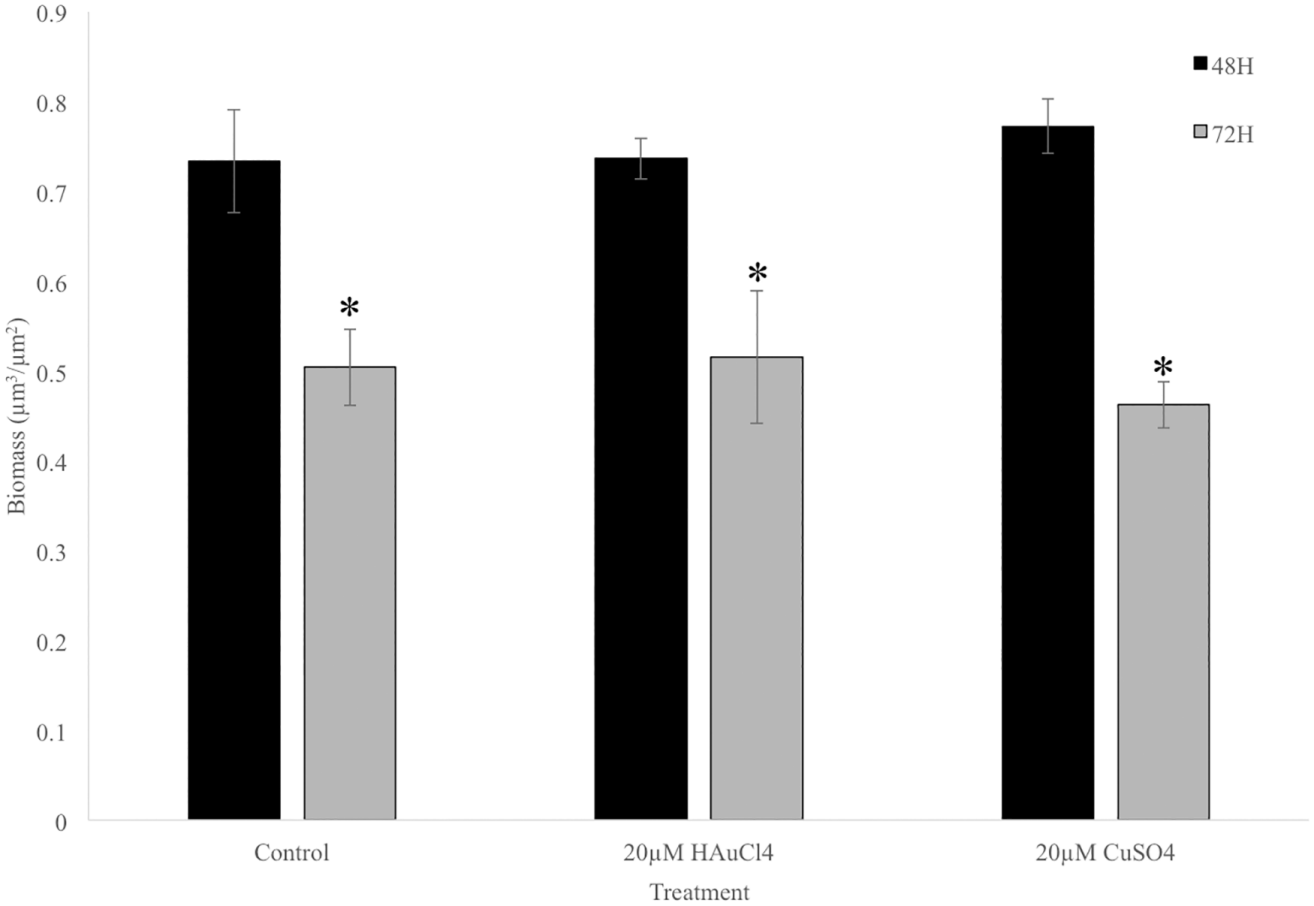
Biomass of control biofilms and biofilms exposed to 20μM HAuCl_4_ or 20μM CuSO_4_ and measured at 48 or 72h. Data presented are from three independent experiments ±SE (p<0.05).

To determine if biomass were responsible for the increase in promoter activity, biofilm biomass was assessed from confocal images using COMSTAT. There were no differences in biomass among control, plus gold, and plus copper samples at each temperature. There was a significant decrease in biofilm biomass in all treatments from 48h to 72h, likely due to the dispersal event typically seen at this stage in *Legionella* biofilm development (Fig 8).

## Discussion

Based on sequenced strains, the GIG operon appears to be rare in the *Legionella* genus, existing in only 3 sequenced strains—Philadelphia 1, Lp02 and SVir, each of which possess the full operon. Multiple homologs of the operon exist in *L*. *pneumophila* at genes *lpg0665-0669*, *lpg0671*, *0673*, *0675*, *0676*, and *lpg2253-2255*. Species within the *Legionella* genus appear to contain multiple paralogous copies of the operon, which group into four distinct clades (Fig 1, Table 1). The *lpg2105-2108* operon in *L*. *pneumophila* Philadelphia 1 is distinct from the others.

The response of the *L*. *pneumophila* GIG operon to HAuCl_4_ and CuSO_4_ is similar to that reported for the GIG operon of *C*. *metallidurans* [16]. At 37°C, planktonic cultures expressed GIG operon promoter activity that increased with the addition of HAuCl_4_ or CuSO_4_ at stationary phase. Conversely, promoter activity was repressed in response to HAuCl_4_ or CuSO_4_ at 26°C (Fig 4). Although not well explored, temperature regulation of genes is not unknown in *Legionella*. Piao, et al., [34] showed that biofilms alter their morphology based on temperature and surface material. Biofilms formed at 37°C and 42°C resembled filamentous mycelial mats, while at 26°C, short, rod shaped bacteria made up the majority of the biofilm. Genes involved in type IV pilus biogenesis and type II protein secretion also showed temperature regulation, with increased expression at 30°C compared to 37°C [35]. The increased HAuCl_4_ sensitivity and opposite expression patterns between 26°C and 37°C in both biofilm and planktonic cultures suggest that temperature regulation may be involved with operon activity. *Legionellae* growing at low-temperatures have a more unsaturated membrane lipids, suggesting that changes in membrane fluidics may also be involved in gold sensitivity if membrane proteins are involved in the response to gold [36]. The *lpg2107* gene appears to possess transmembrane domains and may therefore be localized to the bacterial membrane.

Stationary phase changes in promoter activity in both *Legionella* and *E*. *coli* suggest that known stationary phase regulatory proteins may regulate the operon. *L*. *pneumophila* exhibits unique biphasic gene expression with tight control over exponential vs. stationary phase proteins. Determinants involved in metal or metalloid resistance that are up-regulated after treatment with gold complexes are often controlled by MerR- or ArsR-type regulators, which usually bind “soft” metals or metalloids. In addition to *C*. *metallidurans*, gold response systems exist in *E*. *coli*, controlled through CueR [37] and in *S*. *typhimurium* controlled by GolS [38]. *C*. *metallidurans* also possesses a CupR system that responds to both gold and copper [39]. Homologs of CueR and GolS are found in the *L*. *pneumophila* genome but have not been characterized.

Previous transcriptomic work showed expression of the *Legionella* GIG operon during amoebal infection, suggesting a potential role in virulence [40]. Many *Legionella* virulence genes are regulated by the *rpoS*, *csrA*, and *letA* genes [41–43]. LetA/S activates transmission phase genes, including but not limited to *mip* (macrophage infectivity protein), *dot*/*icm* T4SS, and *flaA* [29, 43, 44–45]. Deletion of *letA* significantly inhibits virulence [29]. However, we saw no changes in promoter activity in Lp02 *ΔletA pGIGgfp* compared to WT Lp02 *pGIGgfp*, suggesting that LetA does not directly regulate expression of the operon. A bioinformatic search for the conserved LetA promoter binding sequence upstream of the GIG operon was also negative, further supporting a lack of direct LetA involvement in operon regulation.

Lp02 *ΔrpoS* cultures showed no response to added HAuCl_4_ or CuSO_4_ when compared to the *ΔrpoS* control. Compared to the Lp02 WT however, promoter activity in *ΔrpoS* strains was significantly repressed (Fig 6). RpoS typically represses motility, infectivity, and cytotoxicity during exponential phase, and up-regulates them during stationary phase [46–47]. It is required for expression of virulence traits and growth within amoebae [41]. RpoS functions in response to stressors in the environment and interacts with other stationary phase regulators such as LetA/S, FliA, *letE*, and LqsR [47–48]. Our data suggest that RpoS may regulate the GIG operon similarly to the way it regulates other stationary phase virulence genes. In fact, regulation may require LetA to release exponential phase repression by RpoS and allow for stationary phase expression, as seen in the regulation of the sRNA molecules RsmYZ [44]. The data support the possibility of multiple regulators for this operon, one for basal expression, and a second for gold/copper response. Investigations into mutants of *fliA* and *letE*, which also coordinate differentiation from replicative phase to transmissive [29,49], as well as *csrA*, a global regulator responsible for the repression of transmission phase genes and activation of replicative phase genes [42], may lead to a better understanding of how this operon is controlled. The recent publication of a *csrA* homolog within the ICE-βox unit–*csrT*–is also a potential regulatory candidate [50].

Biofilms grown at 26°C with 20μM gold or copper showed increased response to the metal ions compared to planktonic cultures at 26°C, with no obvious toxicity. Biomass of biofilms exposed to copper and gold was equivalent to controls (Fig 8). The matrix produced by biofilms that protects bacteria from the effects of toxic metals is not found in planktonic cells. Analyses of operon expression through multiple stages of biofilm development revealed that while baseline expression is present in controls, HAuCl_4_ or CuSO_4_ treatments increase promoter activity, particularly at early developmental stages. Specific up-regulation of the promoter in response to gold occurred at 24h and 72h. Up-regulation early in biofilm development suggests that after the matrix is in place, there is less need for metal response system expression. The up-regulation in response to gold at 72h corresponded with the timing of initial dispersal events in this model (Fig 8). Biofilm dispersal is evident in the difference in biomass between 48 and 72h, but a significant increase in promoter activity is seen at 72h. The up-regulation at this time point may be from cells newly exposed to the external environment.

The GIG operon is present in several other virulent pathogens, including a category B biological agent *Burkholderia pseudomallei*, the causative agent of melioidosis, and a Category A bioterrorism agent, *Francisella tularensis*, the causative agent of tularemia. The presence of this operon in *F*. *tularensis* is particularly interesting since its abundance of pseudogenes and disrupted metabolic pathways are indicative of genome reduction [51–52]. The idea of “use it or lose it” can be applied to this process: genes that are nonessential to the survival of the organism that are mutated or lost. That this operon has been retained suggests it may have an essential function in survival or environmental persistence that could be exploited as a target for antimicrobials.

Many studies have been conducted on the efficacy of water treatment methods on *Legionella*, but few have looked at possible genetic response systems to those treatments, specifically those involved with metal ion response. The controversy over the use of copper/silver ionization as an effective means to protect water systems from *Legionella* colonization may be better explained after the metal response systems in the bacteria are more thoroughly characterized. Lack of information on bacterial response to metals hinders development of effective disinfection strategies. Based on the persistence of *Legionella* in the presence of metal-based disinfection treatments, an increased understanding of biofilm ecology, and in particular the environmental ecology of *Legionella*, is necessary. The results of this study lay groundwork for continued investigation of *Legionella* responses to potentially toxic metal ion concentrations.
